# Potential differences in seed dispersals of low‐height vegetation between single element and windbreak‐like clumps

**DOI:** 10.1002/ece3.5727

**Published:** 2019-10-22

**Authors:** Lin‐Tao Fu

**Affiliations:** ^1^ School of Mechanical Engineering Chengdu University Chengdu China

**Keywords:** accumulative probability, dispersal kernel, release height, vegetation height, vegetation porosity

## Abstract

Wind speed is one of the most important factors for seed wind dispersal. A wind speed reduction region, which could be influenced by vegetation arrangement, will form in the lee of vegetation and therefore affects the seed dispersal. Here, by taking shrub as an example, quantitative differences in seed dispersals of low vegetation between single element and windbreak‐like clumps are numerically investigated. The local variation of stream‐wise wind speed is focused. Empirically parameterized functions of leeward wind distributions are employed. It reveals that the accumulative probability of dispersed seeds from a point source with considering leeward wind reduction could be well fitted by a logistic function. For a fixed release height or vegetation porosity, accumulative probabilities for single element and those for windbreak‐like clumps would intersect at a leeward location. This intersection location decreases linearly with release height but exponentially with porosity. The fitting parameter *r*_0_ (the center of logistic function) for single element increases as the same manner for windbreak‐like clumps, with regard to the increase of release height, porosity, and height. But, the increasing rates for single element are higher than those for windbreak‐like clumps. The fitting parameter *p* (the power index of logistic function) for single element is generally larger than that for windbreak‐like clumps. With the increase of release height, *p* decreases at first but increases then for single element, while it shows opposite trend for windbreak‐like clumps. *p* decreases with porosity for both single element and windbreak‐like clumps. But, the decreasing rate for single element is lower than that for windbreak‐like clumps. *p* increases exponentially with height for windbreak‐like clumps, while it almost keeps constant for single element. These results suggest the potential importance of vegetation arrangement on seed dispersal and therefore possibly provide additional reason for the disagreement among observed dispersal kernels.

## Graphical abstract

By taking shrub as an example, quantitative differences in seed dispersals of low vegetation between single element and windbreak‐like clumps are numerically investigated. Typically, seeds from single element would be transported further than those from windbreak‐like clumps due to the difference in the recovery of leeward wind speed.

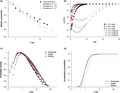

## 1 INTRODUCTION

Seed dispersal plays a very important role in vegetation succession and expansion (Howe & Smallwood, 1982; Travis et al., 2013). Therefore, it is ecologically meaningful to investigate and predict the dispersal of seeds by external driving factors. Wind dispersal is one main dispersal mode for terrestrial vegetations (Bullock et al., 2017; Howe & Smallwood, 1982; Nathan et al., 2011). Wind speed is thus the crucial factor for seed wind dispersal. In open landscapes, the effect of a vegetation element (e.g., a single shrub or tree) on statistically averaged wind speed is negligible. So, theoretical analyses (e.g., Greene & Johnson, 1989; Nathan, Horn, Chave, & Levin, 2002; Nathan et al., 2011) and numerical modeling (e.g., Greene & Johnson, 1996) of seed dispersals for single vegetation element did not consider the change of wind speed around vegetation. In dense vegetation landscapes (e.g., a belt of shrub or forest), vegetation could decrease the wind speed significantly through exchanging momentum with airflow. Therefore, the studies on seed dispersals for dense cases (Nathan et al., 2002, 2011) were conducted by including the decrease of statistically averaged wind speed within vegetation layer, which depends on the plant density or leaf area index (Kaimal & Finnigan, 1994; Raupach, Antonia, & Rajagopalan, 1991).

In fact, the change of local wind speed might be more important for seed wind dispersal. A wind reduction region could form in the lee of vegetation elements (He, Jones, & Rayment, 2017; Leenders, Boxel, & Sterk, 2007; Leenders, Sterk, & Boxel, 2011; Liu, Zheng, Cheng, & Zou, 2018; Mayaud, Wiggs, & Bailey, 2017; Okin, 2008; Raupach, 1992; Yang, Sadique, Mittal, & Meneveau, 2016). Seed will move into this region immediately after being released from vegetation. Greene and Johnson (1996) noticed the effect of local wind reduction from the lee edge of forest to a clearing on seed dispersal. However, for a single vegetation element or patchy, the effect of local wind reduction is usually ignored as mentioned above, particularly for high tree in an open landscape (Greene & Johnson, 1995, 1996). For low vegetation, such as grass, shrub, and low tree (<5 m) with a large crown, the effect of local wind reduction in the leeside may not be ignored, because the averaged wind speed is likely to be more important for seed dispersal than vertical turbulent wind.

Vegetation morphology (or windward shape) could affect the region of local wind reduction due to the difference in drag coefficient (Gillies, Nickling, & King, 2002; Miri, Dragovich, & Dong, 2017). Frontal area ratio (the ratio of height vs. width) is thus introduced to quantitatively parameterize local wind reduction region for a single element in both theoretical and numerical studies (Raupach, 1992; Yang et al., 2016). For field experiments, the effect of the width of vegetation element is, however, likely to be paid less attention. The wind recovery functions in the leeside are usually parameterized by vegetation porosity and height on the basis of measuring data (Leenders et al., 2011; Vigiak, Sterk, Warren, & Hagen, 2003). This is because measurements were commonly conducted for a single element (or clumps) in the cases of frontal area ratio larger than 0.5 (Mayaud et al., 2017). However, vegetation element (or clumps) with low frontal area ratio, for instance, windbreak‐like clumps (many elements standing in a line closely), could be observed in nature, considering the diversity of vegetation arrangement. Comparisons of parameterized leeward wind recovery functions (Mayaud et al., 2017) suggest that remarkable difference in leeside wind speed variation exists between single element and windbreak under identical conditions (vegetation height, porosity, and incoming wind strength). Nevertheless, it is unclear whether this remarkable difference in wind speed could cause considerable change of seed dispersal kernel.

Therefore, the aim of this paper is to investigate the quantitative differences of dispersal kernel between single element and windbreak‐like clumps on open landscape by employing proposed wind recovery functions. The seeds are supposed to be released from a point source. The differences in seed dispersal distributions with release height, vegetation porosity, and vegetation height are quantitatively analyzed. The selected wind recovery functions, master equations for seed motions and other physical parameters are described in Material and Methods section. Main findings are listed in Results section. A concise discussion is arranged at the end of the paper.

## 2 MATERIAL AND METHODS

### 2.1 Leeside wind distribution

**Figure 1:**
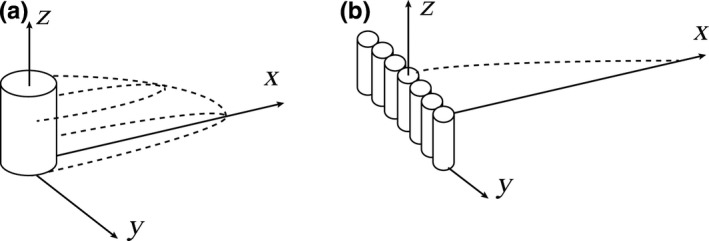
Schematic pictures of wind reduction region in the lee of single element (a) and windbreak‐like clumps (b). In panel (a), the region enclosed by dashed lines is the wind reduction region; in panel (b), the region beneath the dashed line is the wind reduction region

Ideally, open landscape suggests that only one single element or one windbreak stands on a wide flat plane. The interaction among vegetation elements or windbreaks does not exist. Leeside wind distributions for both single element and windbreak‐like clumps are described here. The dominate vegetation type is assumed to be shrub. For convenience, a single vegetation element is simplified as a cylinder (Okin, 2008; Raupach, 1992; Figure 1a). The windbreak‐like clumps are supposed to be an ideal windbreak consisting of multiple cylinder vegetation elements (Figure 1b), which means that the length in *y* direction (perpendicular to stream‐wise direction) is large enough to ignore the edge effect at the two ends. This work is thus mainly focusing on the wind change in the lee of middle location of windbreak.

The description of wind speed in this work could be roughly divided into two parts. The first part includes vegetation itself and the leeward wind speed reduction region. The second part is the remained region where the flow is not disturbed (or the disturbance is so weak that it could be ignored). The horizontal wind speed at any location could be expressed as Equation 1, where *U* (*x*, *y*, *z*) and u_*_ (*x*, *y*, *z*) are time‐averaged horizontal speed and wind shear speed, respectively. *x*, *y*, and *z* are coordinates of horizontal, lateral, and vertical directions, respectively. *κ* is the von Karman's constant and usually taken as 0.41. The aerodynamic surface roughness *z*_0_ is set to be 0.001 m (Raupach et al., 1991). For the undisturbed region (the second part), u_*_ (*x*, *y*, *z*) equals the shear speed of incoming wind, u_*_, by following previous studies (Bullock & Clarke, 2000; Nathan et al., 2002).

(1) $$U\left(x,\;y,\;z\right)=\frac{u_{*}\left(x,\;y,\;z\right)}{\kappa }\ln\left(\frac{z}{z_{0}}\right)$$

(2a) $$u_{*s}\left(x,\;0,\;0\right)=\begin{array}{cc} \frac{\left(u_{*}+u_{*0}\right)}{2}-\frac{\left(u_{*}-u_{*0}\right)x}{D} & if\;-D/2\leq x\ll D/2 \end{array}$$

(2b) $$u_{*s}\left(x,\;0,\;0\right)=\begin{array}{cc} \left(u_{*}-u_{*0}\right)\left[1-\exp\left(-xb/H\right)\right]+u_{*0} & if\;x>D/2 \end{array}$$

(2c) $$u_{*}\left(x,\;0,\;0\right)/u_{*}=1-\exp\left[-C_{\mathrm{Le}}{\left(x/H\right)}^{2}\right]+d_{\mathrm{Le}}\exp\left[-0.003{\left(x/H+e_{\mathrm{Le}}\right)}^{f_{\mathrm{Le}}}\right]$$

For disturbed region (the first part), the description of wind shear speed is divided into two subregions—within vegetation and in the lee of vegetation. There is a lack of data on the wind speed within porous vegetation in atmospheric boundary layer. So, according to previous numerical simulations (Rosenfeld, Marom, & Bitan, 2010) and measurements in subaqueous environment (Chen, Ortiz, Zong, & Nepf, 2012), it is assumed that the ground wind shear speed within a single vegetation decreases linearly from the windward edge to the leeward edge of vegetation element, in the form of half‐ellipse contour. Several basal shapes of wind reduction region (triangle, rectangular, and half‐ellipse) in the lee of plants (Leenders et al., 2011; Okin, 2008; Raupach, 1992) have been proposed. Recent observations (Leenders et al., 2011; Mayaud et al., 2017) and simulations (Sadique, Yang, Meneveau, & Mittal, 2017; Yang et al., 2016), however, indicated that the half‐ellipse shape proposed by Leenders et al. (2011) is likely to be more reasonable for porous shrub vegetation element. The semiminor axis of the half‐ellipse is set to be *D*/2. The maximum stream‐wise length (*L_x_*) of wind reduction region (the semimajor axis) is about 7.5*H* (Leenders et al., 2011). Leenders et al. (2011) assumed that the wind shear speed recovers exponentially with the leeward distance from the leeward edge of vegetation element to the maximum stream‐wise length, also in the form of half‐ellipse contour. The change of ground shear wind speed along the central line in the first part for single element vegetation could thus be expressed as Equation 2. Here, according to Mayaud et al. (2017), u_*0_ = (1.46 × *θ *− 0.4076)u_*_, *b* = 1.05 × *θ *+ 0.1627, where u_*0_ is the lowest value of wind shear speed at the leeward edge of vegetation element, and *θ* is the vegetation porosity. In the case of windbreak, the change of ground wind shear speed within windbreak could also be described as Equation 2a, while the ground wind shear speed in the lee of windbreak is expressed by Equation 2c (Vigiak et al., 2003). *C*_Le_ = 0.008 – 0.17*θ* + 0.17*θ* ^1.05^, *d*_Le_ = 1.35 exp (−0.5*θ* ^0.2^), *e*_Le_ = 10 (1 – 0.5*θ*), and *f*_Le_ = 3 − *θ*. Finally, for the continuity of wind shear speed at the interface between wind reduction region and upper undisturbed region, the value of wind shear speed within wind reduction region is supposed to grow up linearly, from ground wind shear speed to incoming wind shear speed u_*_, with the increase of vertical coordinate *z*.

The atmospheric turbulence is included here for trajectory calculation. The instantaneous wind could be written as ***u*** = ***U*** + ***u*′**, where ***U*** is the time‐averaged speed, and the prime represents a fluctuating speed. Here, the time‐averaged speeds in vertical and lateral directions are set to be zero. The variations of turbulent fluctuations along trajectory could be described statistically by Equation 3 (Kok & Renno, 2009; Van Dop, Nieuwstadt, & Hunt, 1985). *n*_G_ is a Gaussian distributed random number with zero mean and unit standard deviation. *σ* ***_u_*** is the standard deviation of wind fluctuation. Based on previous study (Nishimura & Hunt, 2000), detailed values in three directions are *σ_u_* = 2.5u_*_ (*x*, *y*, *z*), *σ_v_ *= *σ_w_* = 1.3u_*_ (*x*, *y*, *z*), where *u*, *v*, and *w* are horizontal, lateral, and vertical directions. The Lagrangian timescale and the time‐averaged turbulent dissipation rate are defined as *T_l_* = 2*σ_w_* ^3^/(*C*_0_ *ε*_0_) and *ε*_0_ = u_*_ (*x*, *y*, *z*)^3^/*κz*, respectively. *C*_0_ could be taken as 4.0. These settings are based on four reasons below. First, the main concern of this work is the local change of horizontal wind speed. Second, the release points are located in the stream‐wise central line where the impact of lateral wind should be the weakest. Third, measurements revealed that horizontal and vertical turbulences gradually recover in accordance with the recovery of horizontal wind speed (Hagen & Skidmore, 1971). Fourth, the impact of vertical speed on particle motion would be much weaker than that of horizontal speed in the case of shrub vegetation. Since shrub height is typically lower than 5 m in atmospheric boundary layer, effective vertical and horizontal speeds for seed dispersal are much smaller than those in the case of trees (typical heights ranging from 10 to 30 m). Measurements suggested that averaged vertical speed could be about several percents of horizontal speed (Hagen & Skidmore, 1971), and vertical turbulence was possibly much smaller than horizontal turbulence (Mayaud, Wiggs, & Bailey, 2016) as well as terminal depositing velocity of seeds.

(3) $$u^{\prime }\left(t+dt\right)-u^{\prime }\left(t\right)=-u^{\prime }\left(t\right)dt/T_{l}+n_{G}\sigma_{u}\sqrt{2dt/T_{l}}$$

### 2.2 Master equation of seed motion

The motion of seeds could be usually driven by multiple forces (Maxey & Riley, 1983); however, only the gravity and the drag are considered here. The translational motion of seeds could thus be determined by Equation 4. ${{\;}^{⇀}\;\;\;\;x}_{p}$ is the location of seeds, ***g*** the gravitational acceleration, and *ρ*_s_ the seed density. *C*_D_ is the drag coefficient and defined as *C*_D_ = [(32/Re_D_)^2/3^ + 1]^3/2^ for irregular particles (Cheng, 1997). Re_D_ = *ρ*_a_ *d*_s_ |${{{\;}_{V}\;\;\;\;}^{⇀}}_{\;\;r}$|/*μ*, where *ρ*_a_ is the air density, *d*_s_ the averaged diameter of seeds, *μ* the dynamic viscosity of air, and ${{{\;}_{V}\;\;\;\;}^{⇀}}_{\;\;r}=\left(\frac{d{{\;}^{⇀}\;\;\;\;x}_{p}}{\mathrm{dt}}-u\right)$.

(4) $$\frac{d^{2}{{\;}^{⇀}\;\;\;\;x}_{p}}{dt^{2}}=-0.75\frac{\rho_{a}C_{D}}{\rho_{s}d_{s}}\left(\frac{d{{\;}^{⇀}\;\;\;\;x}_{p}}{\mathrm{dt}}-u\right)\left|\frac{d{{\;}^{⇀}\;\;\;\;x}_{p}}{\mathrm{dt}}-u\right|-g$$

### 2.3 Other settings

According to previous studies (Bullock & Clarke, 2000; Leenders et al., 2011; Mayaud et al., 2017), some selected physical constants are listed in Table 1. The initial speeds of released seeds are set to be zero. Fourth‐order Runge‐Kutta method is employed to numerically predict the trajectories of seeds. The trajectory‐crossing effect on the Lagrangian timescale (Arritt et al., 2007; Csanady, 1963) is considered. Different from previous investigations (Arritt et al., 2007; Wilson, 2000) in which the terminal settling velocity was employed, the relative speed between particle and flow is adopted to depict the trajectory‐crossing effect (Anderson, 1987), as shown in Equation 5, where *β* = 1.0 (Kok & Renno, 2009). The discrete time step for trajectory calculation is thus determined as *dt* = 0.01 × min (*T_pz_*, *T_py_*, *T_px_*).

(5) $$T_{\mathrm{pz}}=\frac{T_{l}}{\sqrt{1+{\left(\beta V_{r}/\sigma_{w}\right)}^{2}}};\;T_{\mathrm{py}}=\frac{T_{l}}{\sqrt{1+{\left(\beta V_{r}/\sigma_{v}\right)}^{2}}};\;T_{\mathrm{px}}=\frac{T_{l}}{\sqrt{1+{\left(2\beta V_{r}/\sigma_{u}\right)}^{2}}}$$

**Table 1:** Some physical constants used in this work

*H*/*D*	*d*_s_ (mm)	*ρ*_s_ (kg/m^3^)	*ρ*_a_ (kg/m^3^)	*μ* (kg/m/s)	*g* (m/s^2^)
0.5	0.5	500	1.225	1.78 × 10^–5^	9.81

**Figure 2:**
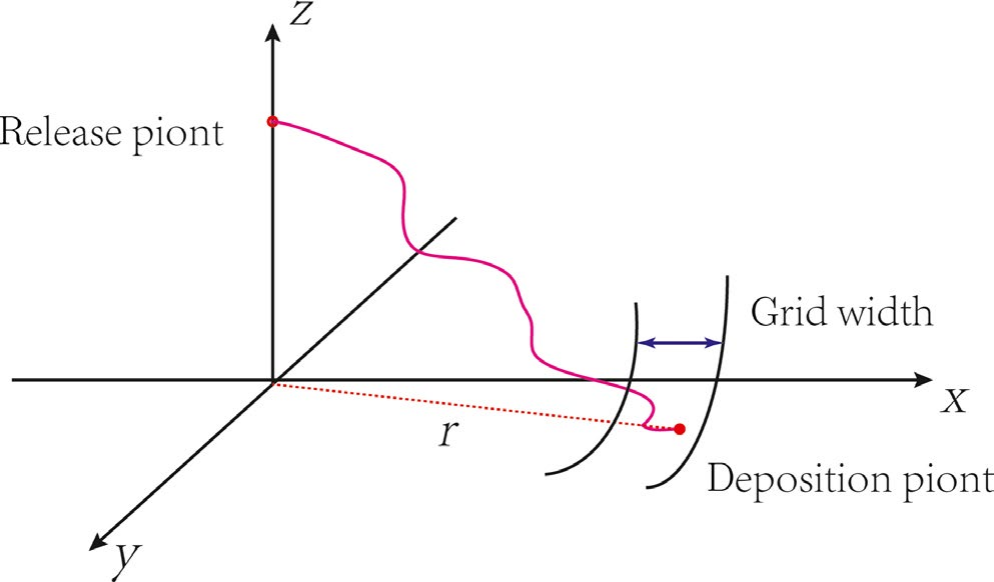
Schematic pictures for grid setting and the collection of dispersed seeds

All seeds are released at a point source from mother plant. To make the results more meaningful, 10^6^ seeds are released in each defined calculating condition. As previous study did (Bullock & Clarke, 2000), grids for information statistics are set along radial direction from the central location of vegetation element (Figure 2). Total of 100 discrete grids are applied for common cases. The intervals of the first, second, third, forth, and fifth ten grids are 0.1*H*, 0.2*H*, 0.3*H*, 0.4*H*, and 0.5*H*, respectively. The interval of remained grids is 1*H*. The distance from seeds' deposition location to vegetation center (being represented by “*r*”) is used to judge in which seeds are located. The distribution function and accumulative probability of dispersed seeds are then calculated and analyzed grid by grid. To remind the role of the release threshold wind in seed dispersal (Schippers & Jongejans, 2005), the lowest wind shear speed that could cause seed release is assumed to be 0.2 m/s, by considering the height of vegetation element used in this work. The probability of incoming wind speed follows a Weibull distribution law. The accumulative probability of incoming wind speed could be expressed as $f\left(u_{*};\;k,\;c\right)=1-\exp\left[-{\left(u_{*}/c\right)}^{k}\right]$, where *k* = 2 (Seguro & Lambert, 2000), and thus, $c=2u_{*c}/\sqrt{\pi }$. u_*_ *_c_* is the averaged wind shear speed and taken as 0.35 m/s here with a cut‐off maximum shear speed u_*_ = 0.65 m/s. Nine release heights (*H*_0_ = 1.0*H*, 0.9*H*, 0.8*H*, 0.7*H*, 0.6*H*, 0.5*H*, 0.4*H*, 0.3*H*, and 0.2*H*) are involved below. When the effect of local wind reduction is not considered, the vegetation porosity is equal to 1. Five values of porosity are 0.3, 0.4, 0.5, 0.6, and 0.7, respectively, on the basis of recent field observations (Mayaud et al., 2017). Besides, five vegetation heights, *H* = 0.5, 1.0, 1.5, 2.0, and 2.5 m, are employed. More detailed information about the simulations and the numerical code could be found in Appendix S1. Moreover, the observed data for *Calluma* in Table 2 in the work of Bullock and Clarke (2000) are applied to verify the numerical model. From their work, the size and density of seeds are about 0.58 mm and 225 kg/m^3^, respectively. The averaged release height *H*_0_ is 0.144 m. Because they did not show the wind speed directly (Katul et al., 2005), a long‐time averaged wind shear speed u_*_ *_c_* = 0.30 m/s (which corresponds to the speed of 6.74 m/s at height *H* = 10 m; following a Weibull distribution law) is assumed. Another point required for numerical model is the vegetation porosity. However, there is no idea about the porosity in the study of Bullock and Clarke (2000). Therefore, three possible porosities (*θ* = 0.3, 0.5, and 0.7) are selected. Because the total release number of seed source in field could not be accurately determined, the relative proportion of seed deposition is employed instead of seed density per area for comparison. The relative proportion at each focused location is calculated as the gathered seed number at each focused location versus the total gathered seed number from all focused locations.

## 3 RESULTS

### 3.1 Preliminary comparison

**Figure 3:**
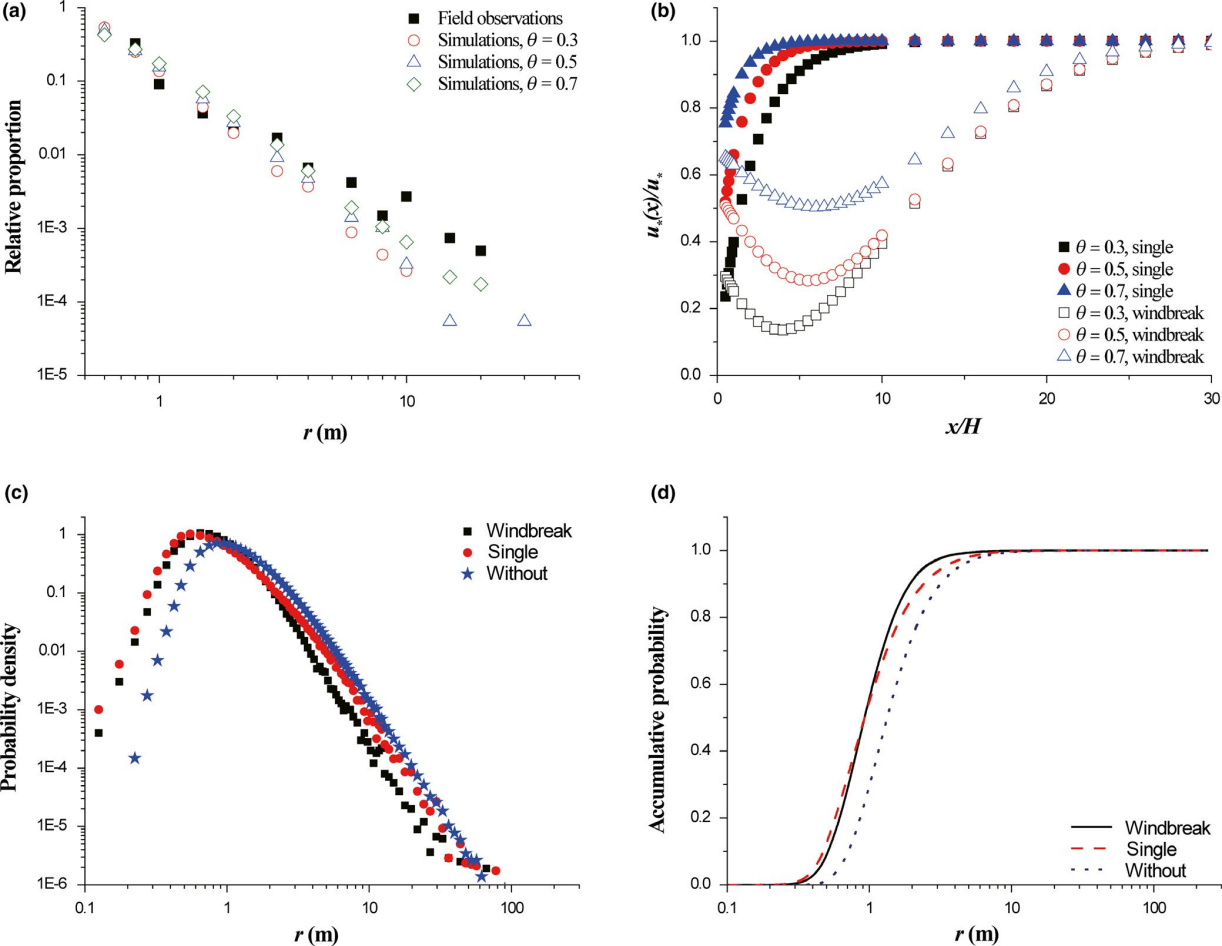
Relative proportion of seed deposition (a), dimensionless wind shear speed u_*_(*x*)/u_*_ (b), probability density of seed deposition (c), and accumulative probability of seed deposition (d) in the lee of vegetation. In panel (a), solid scatters are observed data in Table 2 from Bullock and Clarke (2000), open scatters are simulated data by averaged wind shear speed u_*_ *_c_* = 0.3 m/s. “without” suggests seed dispersal from a point source without considering effects of both single element and windbreak on wind speed. In panels (c) and (d), *H* = 0.5 m, *H*_0_ = 0.5*H*, *θ* = 0.5, and u_*_ *_c_* = 0.35 m/s

Above all firstly, the numerical model was tested by field observation (Figure 3a). It could be found that under reasonable setting for wind speed, the numerical model could well reproduce the observation data in Table 2 of Bullock and Clarke (2000). Also, vegetation porosity could affect the relative proportion of seed deposition after dispersal (open scatters in Figure 3a), because the evolution of wind speed in the lee of vegetation would be influenced by vegetation porosity (Figure 3b). Typically, the recovery rate of leeward wind speed increases with the increase of vegetation porosity. Figure 3b reveals that there is a significant difference in leeward wind speed evolution between single element and windbreak. For a fixed value of vegetation porosity, wind speed in single element case is generally larger than that in windbreak case. Then, the deposition patterns of dispersed seeds between without considering wind reduction effect and with considering wind reduction effect (single element case and windbreak case) are compared (Figure 3c,d). It could be seen that considering effects of wind reduction could evidently increase the seed deposition near source. For example, the accumulative probability with considering wind reduction at *r* = 1.0 is about 0.55, while the probability without considering wind reduction at *r* = 1.0 is about 0.30 (Figure 3d). Generally, the difference in wind speed recovery behind both single element and windbreak could not change the deposition pattern (unimodal distribution of probability density) of seeds released from a point source (Figure 3c). It could be found that the quantitative difference of seed dispersal kernels between single element and windbreak in the lee of vegetation (*r* > *D*/2) is visible. When *r* is <2 m, the probability density in the case of windbreak is higher than that of single element. However, when *r* is larger than 2 m, the density in the case of windbreak turns to be smaller than that of single element. The quantitative difference of seed dispersal between the two cases is also clearly shown by the accumulative probability (Figure 3d). The accumulative probabilities of two cases intersect at *r* around 1 m. When *r* is smaller than 1 m, the accumulative probability of single element is higher than that of windbreak. When *r* is larger than 1m, the accumulative probability of single element is lower than that of windbreak. Further data analyses show that the accumulative probabilities of both cases under our ideal settings could be well expressed by logistic curve (Equation 6; *R* ^2^ > .98), where *A*_1_ = 0, *A*_2_ = 1, *r*_0_ is the central location (inflection point here defined as the distance corresponding to accumulative probability 50%), and *p* is the power index (Hill's slope). The two fitting parameters, *r*_0 _and *p*, are practical proxy for quantitatively evaluating the difference in seed dispersals between single element and windbreak.

(6) $$\text{Fap}=\frac{A_{1}-A_{2}}{\left[1+{\left(r/r_{0}\right)}^{p}\right]}+A_{2}$$

### 3.2 Impact of seed release height

**Figure 4:**
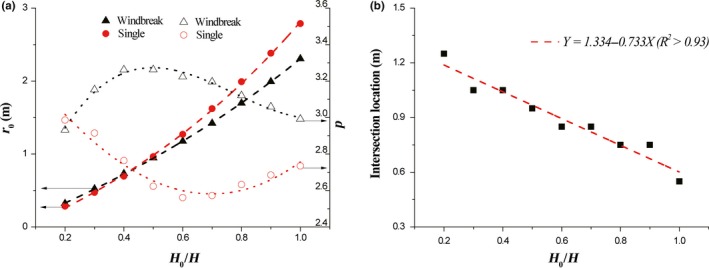
The impacts of release height on fitting parameters (a) and intersection location (b). *H* = 0.5 m, *θ* = 0.5, and u_*_ *_c_* = 0.35 m/s

Simulated results show that the dispersal kernels of seeds released from different heights could be expressed by Equation 6. The two fitting parameters of these results are shown in Figure 4a. It could be found that the central location *r*_0_ increases with the increase of release height in both cases. Although the values of *r*_0_ for single element are lower than those for windbreak below *H*_0_/*H* = 0.4, but they turn to be greater than those for windbreak above *H*_0_/*H* = 0.4, because a higher increase rate leads to quick increment of *r*_0_ for single element. Quantitative analyses reveal that the variation of *r*_0_ with release height for windbreak under current settings could be described as *r*_0_ = −2.24 + 2.23 exp (0.71*H*_0_/*H*) (*R* ^2^ > .99) and that for single element is *r*_0_ = −1.77 + 1.66 exp (1.02*H*_0_/*H*) (*R* ^2^ > .99). Significant difference in variations of power index *p* between windbreak and single element is shown. Generally, the values of *p* for windbreak are larger than those for single element. With the increase of release height, the values of *p* for windbreak increase firstly but decrease then after reaching a peak, while opposite change law occurs for single element. The variations of *p* with release height for windbreak and single element are *p* = 2.19 + 5.19 *H*_0_/*H*−7.67 (*H*_0_/*H*)^2^ + 3.30 (*H*_0_/*H*)^3^ (*R* ^2^ > .92) and *p* = 3.42 – 2.29 *H*_0_/*H* + 1.41 (*H*_0_/*H*)^2^ + 0.22 (*H*_0_/*H*)^3^ (*R* ^2^ > .92), respectively. Besides, it reveals that the intersection location decreases linearly with the increase in release height (Figure 4b).

### 3.3 Impact of vegetation porosity

**Figure 5:**
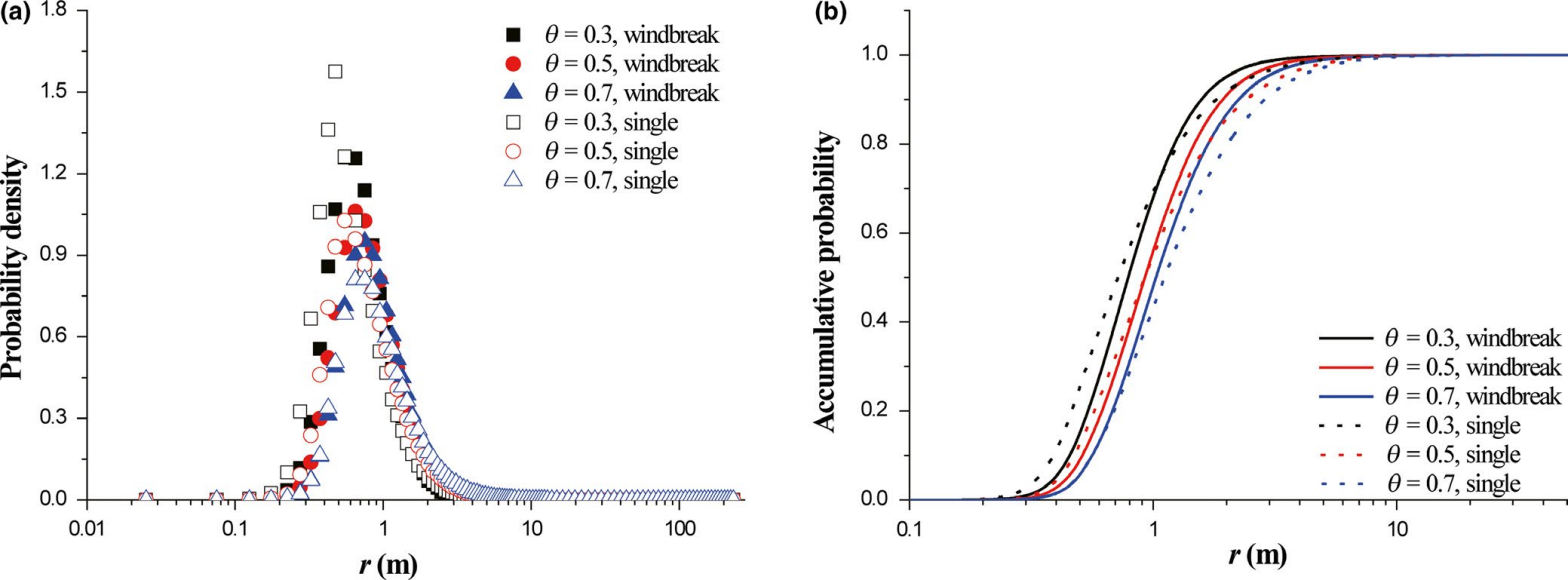
The variations of probability density (a) and accumulative density (b) with vegetation porosity. *H* = 0.5 m, *H*_0_ = 0.5*H*, and u_*_ *_c_* = 0.35 m/s

**Figure 6:**
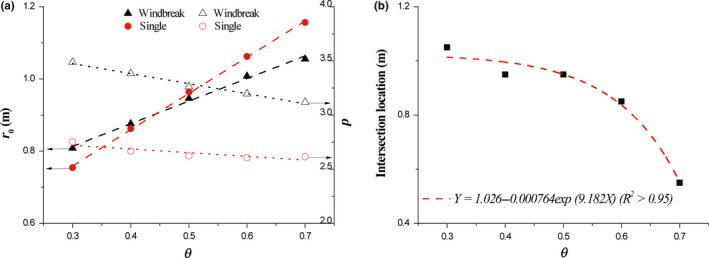
The impacts of vegetation porosity on fitting parameters (a) and intersection location (b). *H* = 0.5 m, *H*_0_ = 0.5*H*, and u_*_ *_c_* = 0.35 m/s

The change of vegetation porosity does not alter the distribution pattern of deposited seeds (Figure 5). Typically, the transport distance of seeds increases with the increase of vegetation porosity. In comparison to windbreak, probability density for single element is more sensitive to the variation of porosity (Figure 5a). With the increase of porosity, *r*_0_ increases linearly for both windbreak and single element (Figure 6a). The variation of *r*_0_ with porosity for windbreak case could be described as *r*_0_ = 0.63 + 0.63*θ* (*R* ^2^ > .99) and that for single case is *r*_0_ = 0.46 + 1.00*θ* (*R* ^2^ > .99). Oppositely, *p* decreases linearly with the increase of porosity for both cases (Figure 6a). The variation of *p* with *θ* for windbreak case could be described as *p* = 3.75 – 0.93*θ* (*R* ^2^ > .99) and that for single case is *p* = 2.82 – 0.34*θ* (*R* ^2^ > .99). The intersection point of accumulative probabilities between windbreak and single element always exists with the variation of porosity (Figure 5b). Further data analysis shows that intersection location exponentially decreases with porosity (Figure 6b).

### 3.4 Impact of vegetation height

**Figure 7:**
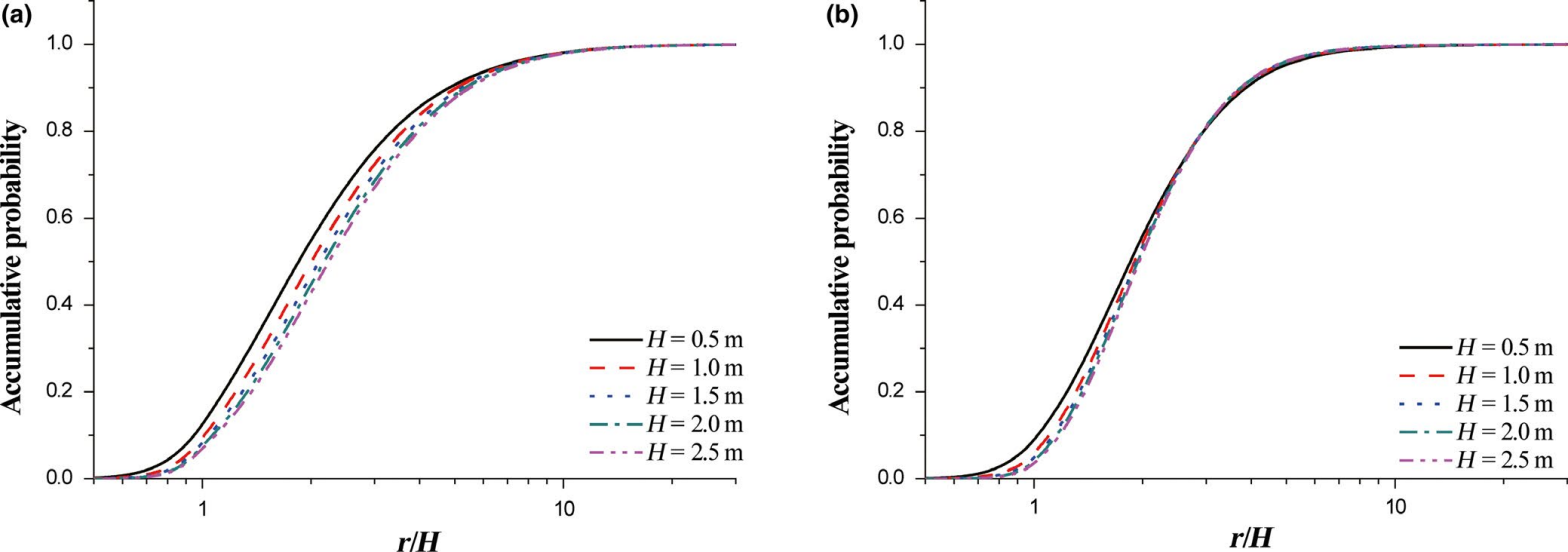
Accumulative probabilities of single element (a) and windbreak‐like clumps (b) versus dimensionless leeward distance. *H*_0_ = 0.5*H*, *θ* = 0.5, and u_*_ *_c_* = 0.35 m/s

**Figure 8:**
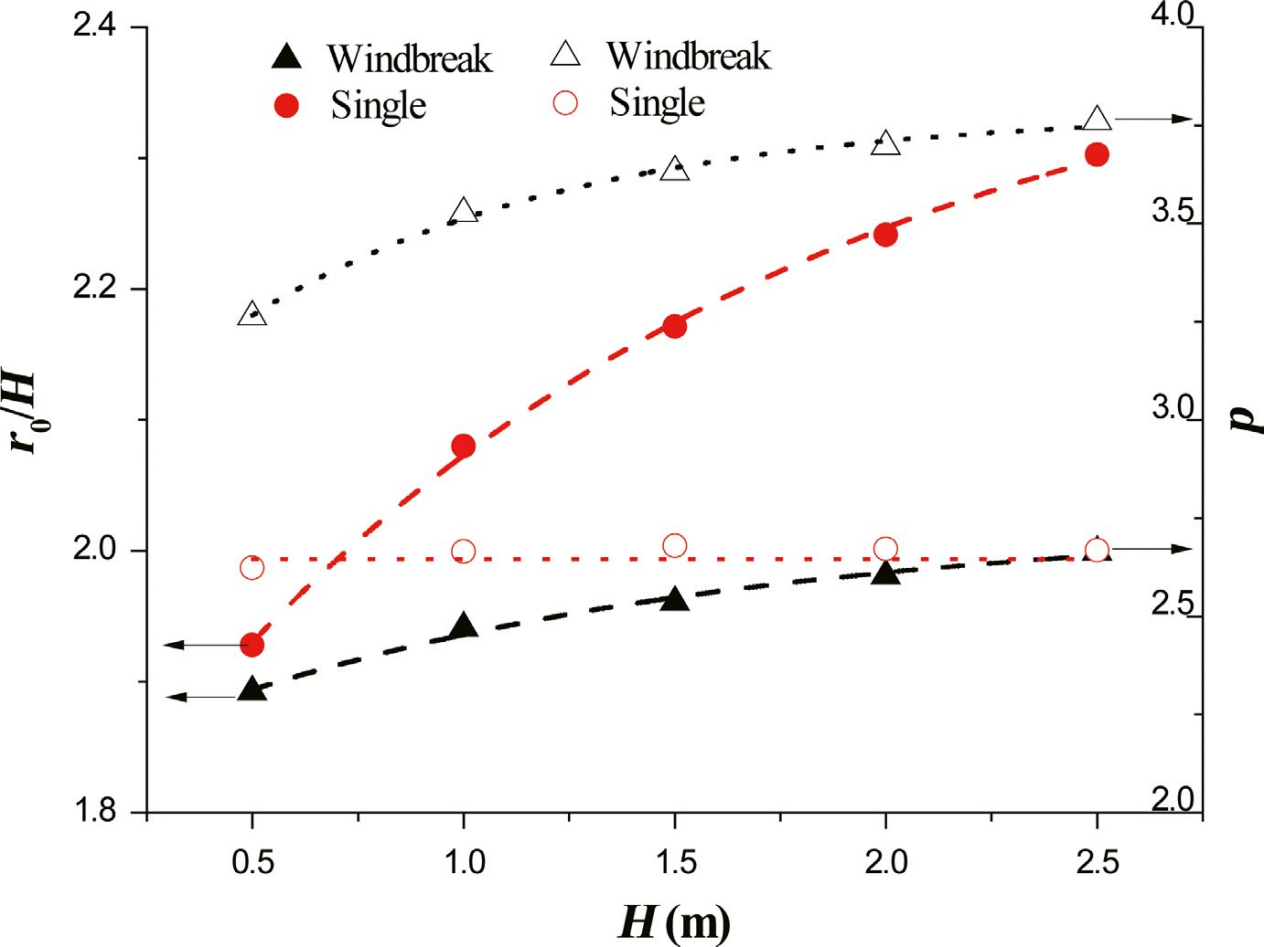
The impacts of vegetation height on fitting parameters. *H*_0_ = 0.5*H*, *θ* = 0.5, and u_*_ *_c_* = 0.35 m/s

Simulated results suggest that the vegetation height could also affect the deposition of seeds in the lee of vegetation (Figure 7). To understand the impact of vegetation height better, the dimensionless leeward distance *r*/*H* is employed. No intersection of accumulative probability curve occurs for single case with the change of vegetation height (Figure 7a). With the increase of vegetation height, the accumulative probabilities for windbreak case intersect at *r*/*H* = 3, where the accumulative probability is around 0.8 (Figure 7b). The accumulative probability distributions are then fitted by Equation 6. And, the variations of the two key parameters with vegetation height are shown in Figure 8. It is easily understood that *r*_0_ increases with vegetation height. However, it could be seen that the values of *r*_0_/*H* for both cases show non‐linear responses to vegetation height. The variation of *r*_0_/*H* for windbreak case could be described as *r*_0_/*H* = 2.24 – 0.19 exp (−0.79*H*) (*R* ^2^ > .98) and that for single case is *r*_0_/*H* = 2.43 – 0.70 exp (−0.68*H*) (*R* ^2^ > .98). The responses of *p* to vegetation height between windbreak case and single case are different. For windbreak case, *p* increases exponentially with vegetation height. Namely, *p* = 3.79 – 0.99 exp (−1.28*H*) (*R* ^2^ > .99). However, for single case, *p* keeps a constant about 2.66.

## 4 DISCUSSION

Wind dispersal is one of the most important seed dispersal modes for terrestrial vegetation (Bullock et al., 2017; Howe & Smallwood, 1982; Nathan et al., 2011). The wind speed is therefore the key factor determining the motion of seeds. The presence of vegetation will alter the wind distribution nearby and form a wind reduction region in the lee of vegetation (Mayaud et al., 2017; Okin, 2008; Raupach, 1992). And, the arrangement of vegetation could affect the reduction of wind speed in the leeside (Liu et al., 2018). This work focused on the quantitative difference in dispersal kernels of seeds caused by different wind reductions owing to vegetation arrangement (single element and windbreak‐like clumps). The simulated results suggest that the accumulative probability distributions of deposited seeds could be expressed by logistic curve. The two parameters of logistic curve, the central location *r*_0_ and power index *p*, are employed to quantitatively evaluate the difference in seed dispersals between single element and windbreak‐like clumps.

Comparison studies (Figure 3b) suggested that under identical physical settings, (a) the lowest wind speed in the leeside for windbreak‐like clumps is smaller than that for single element, and (b) the wind speed in leeward wind reduction region for single element recovers much faster than that for windbreak‐like clumps (Mayaud et al., 2017). Therefore, the responses of *r*_0_ to involved factors (release height, vegetation porosity, and vegetation height) for single element are more sensitive to those for windbreak‐like clumps. This is because *r*_0_ represents the transport distance of whole seeds, which is dominated by both leeward wind speed and its recovery. In contrast, the values of *p* for windbreak case are typically higher than those for single case. This is because *p* is a slope factor that describes the steepness of the accumulative probability curve and therefore reflects the degree of the spread of deposited seeds. Low wind speed and the corresponding low level of turbulence for windbreak‐like clumps result in fast deposition and low degree of the spread, which suggests a high value of *p*. As shown in Figure 3b, the wind reduction region for single element is smaller than that for windbreak‐like clumps. For windbreak‐like clumps, seeds always travel within wind reduction region by experiencing wind reduction at first and wind increase then. The values of *p* for windbreak thus increase firstly but decrease then after reaching a peak. For single element, seeds could have an opportunity to travel out of wind reduction region (where wind speed is large) at first and then fall in wind reduction region (where wind speed is small). The *p* values for single element thus decrease firstly but increase then after reaching a low. The increase of vegetation porosity suggests the increase of wind speed in the leeside, which therefore leads to the decrease of *p*. Besides, for most of terrestrial vegetation, porosity will vary with season change. A larger decrease rate of *p* indicates that windbreak‐like clumps would be more sensitive to season change than single element. Finally, the difference in responses of *p* to vegetation height between single element and windbreak‐like clumps suggests the importance of vegetation height in seed dispersal. However, it should be reminded that all results shown here for single element are based on the ratio of *H*/*D* = 0.5. The wind reduction region in the lee of single element is also affected by the ratio of *H*/*D* (Raupach, 1992; Sadique et al., 2017; Yang et al., 2016). Therefore, more simulations or field observations are further required to determine the effect of the ratio of *H*/*D* on wind reduction region and then on seed dispersal for single element. Another important issue is the assumption about vertical wind speed in this work. Previous studies indeed suggested the importance of vertical turbulence in seed wind dispersal in the cases of heterogeneous canopy conditions (Bohrer, Katul, Nathan, Walko, & Avissar, 2008; Damschen et al., 2014; Nathan et al., 2011). The results shown here could thus provide partial contribution to promote the understanding of seed dispersal for high canopy condition (e.g., forest). A comprehensive study on the seed dispersal of height vegetation requires further parameterizations in the variations of both averaged speed and turbulence (particularly for vertical component) along with height.

Furthermore, above findings are obtained on the basis of some ideal settings. Nevertheless, seed wind dispersal is affected by multiple factors apart from wind speed. Under identical wind speed, seeds could be accelerated differently due to diverse seed traits (e.g., mass density, size, and shape). From a viewpoint of modeling, initial and boundary conditions for seeds (e.g., vegetation architecture, seed source distribution on vegetation, seed release threshold, and so on) are also very important (Cousens, Hughes, & Mesgaran, 2018). Recent study (Johansson, Lönnell, Rannik, Sundberg, & Hylander, 2016) indicated that air humidity (as an external driving factor) could affect the water content of seeds, which therefore alters the release threshold of seeds. Therefore, with efforts on these aspects, we could better understand the quantitative difference in seed dispersal among various vegetation arrangements.

## CONFLICT OF INTEREST

The author declares no conflict of interest.

## AUTHOR CONTRIBUTIONS

LTF designed the study, collected and analyzed the data, and wrote the manuscript.

## Data Availability

All employed data are available in Supporting Information (Appendices S1 and S2).
