# Supplementary material for: Potential differences in seed dispersals of low‐height vegetation between single element and windbreak‐like clumps
Source: Ecol Evol. 2019 Oct 22;9(22):12639–48. doi: 10.1002/ece3.5727 (PMC6875573; doi:10.1002/ece3.5727)
Supplement: Supplementary file 1 [file ECE3-9-12639-s001.docx]

**Appendix S1**

The numerical code is written in Fortran language (FOR77). Forward difference is employed to conduct the discretization of Equation (4) in main text. The variable trajectory of each seed is recorded step by step till the seed deposits. For a better understanding or further implementation, I show the flow chart of my simulations (Figure S1) and the original FOR77 code (Figure S2) for windbreak cases below. However, because of the length of code, the code is shown in the form of pictures (this also is the best way for readers to understand the code). Please contact me if you have any questions about the code shown below.

**Figure S1**


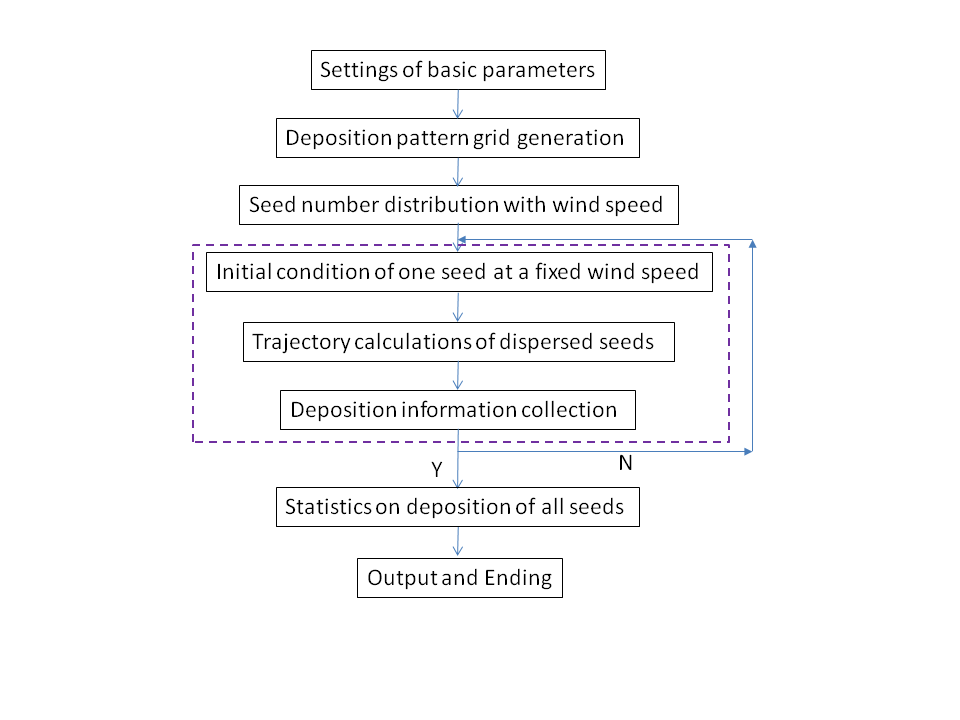


Figure S1. The flow chart for numerical simulations of seed dispersal responding to the original Fortran code. If all the prepared seeds deposit on surface successfully (denoting as “Y” in the figure), the program goes on the way to end. If at least one seed doesn’t deposit on surface (denoting as “N” in the figure), the program goes along the arrow to continue the iteration.

**Figure S2**


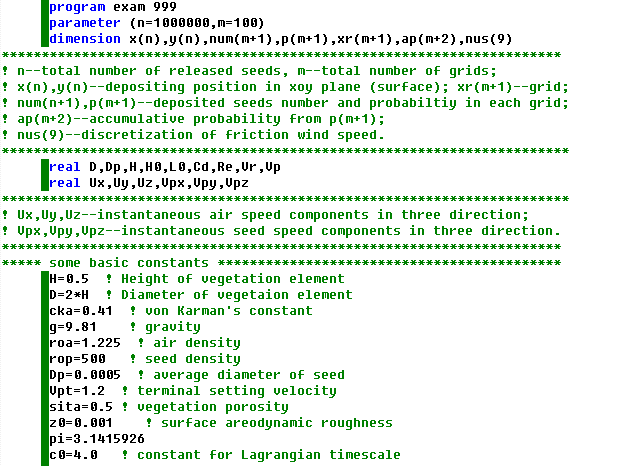


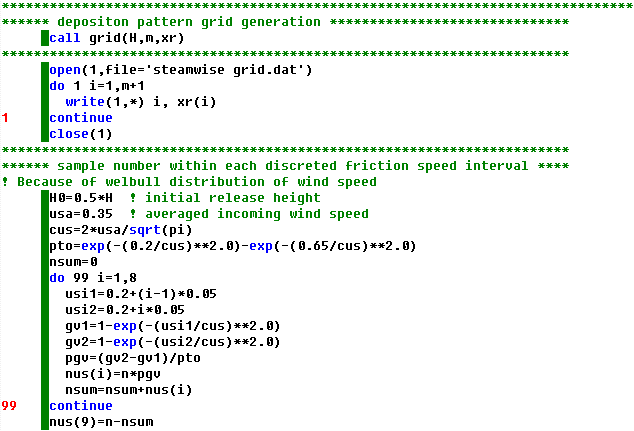


Figure S2. The original FOR code for seed dispersal simulations for windbreak cases.


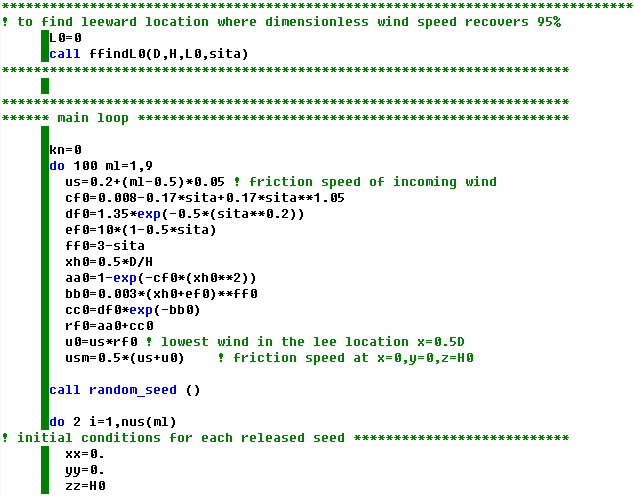


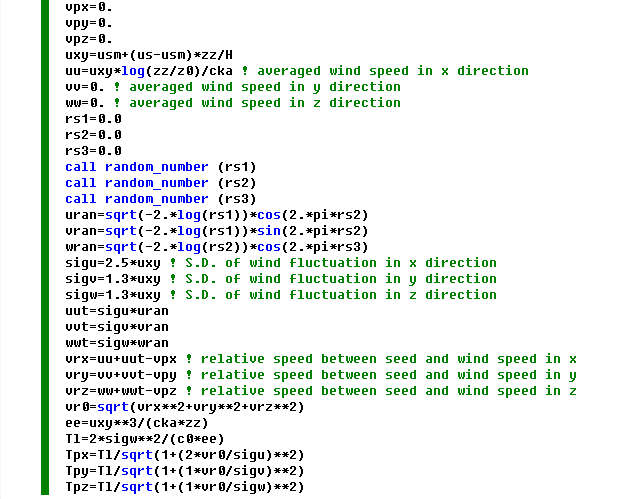


Figure S2 (continued).


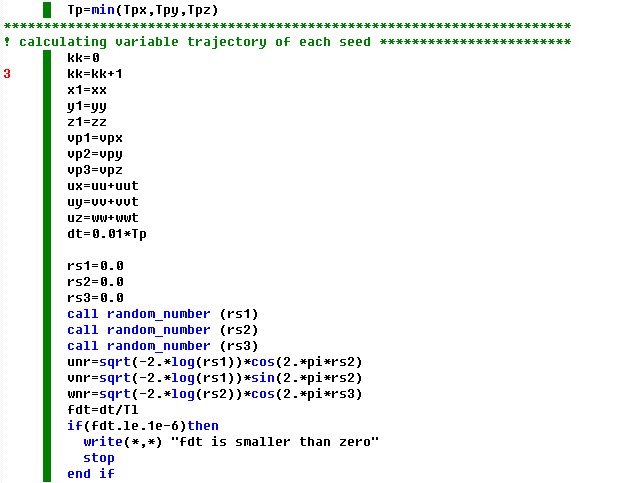


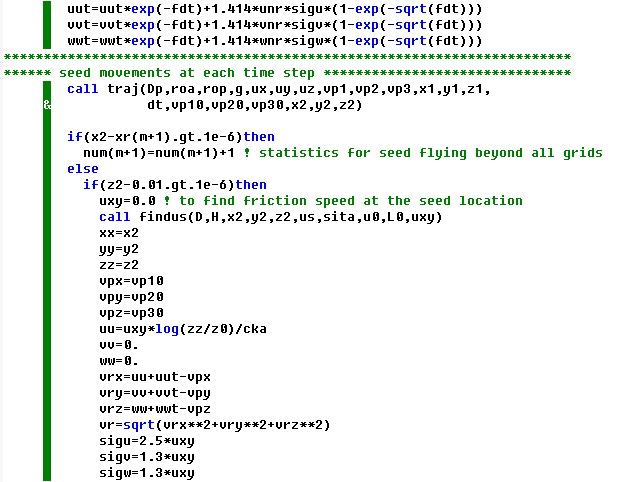


Figure S2 (continued).


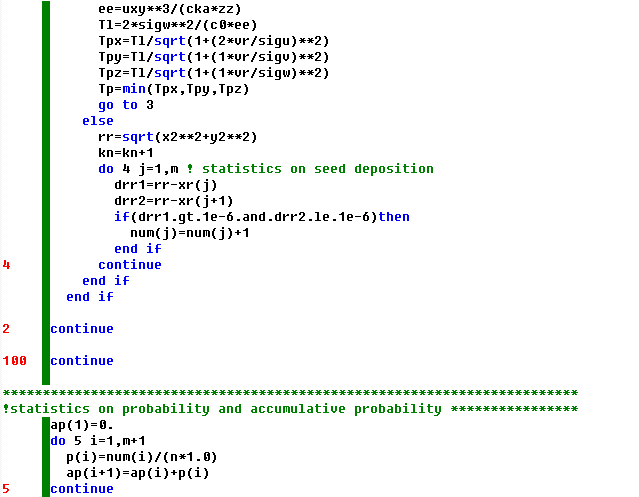


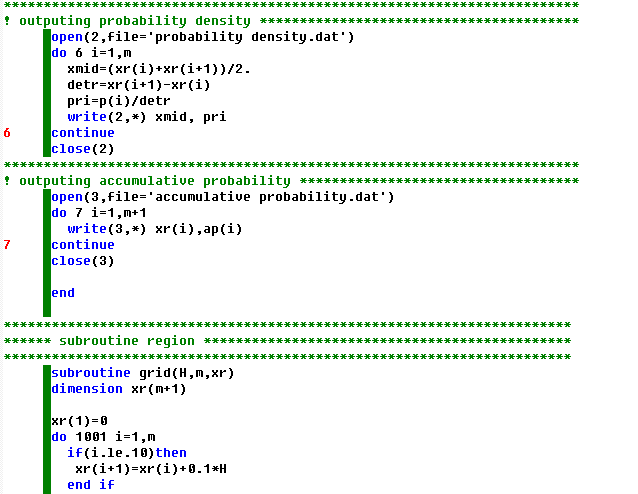


Figure S2 (continued).


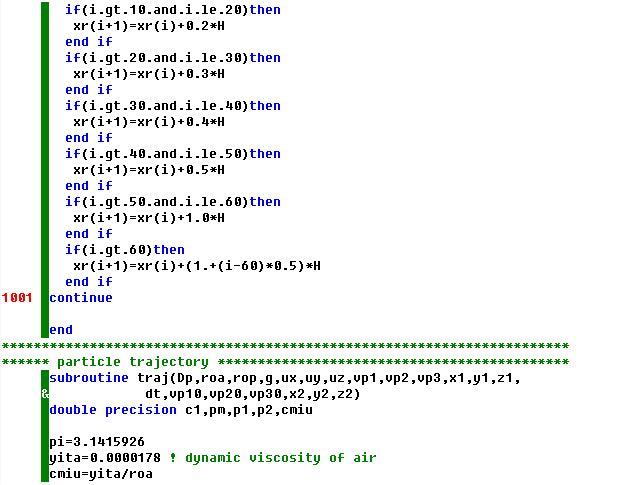


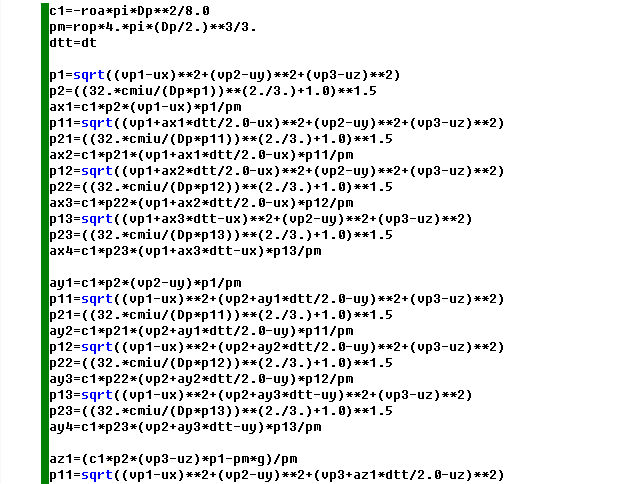


Figure S2 (continued).


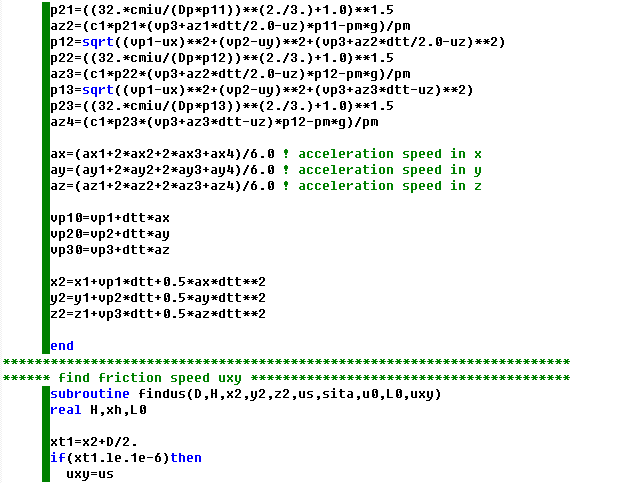


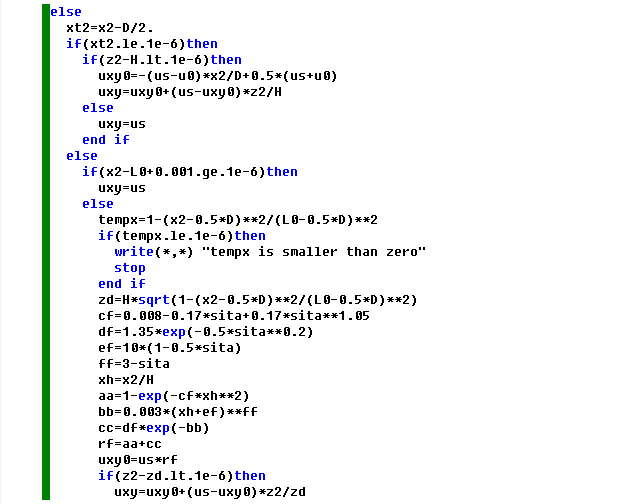


Figure S2 (continued).


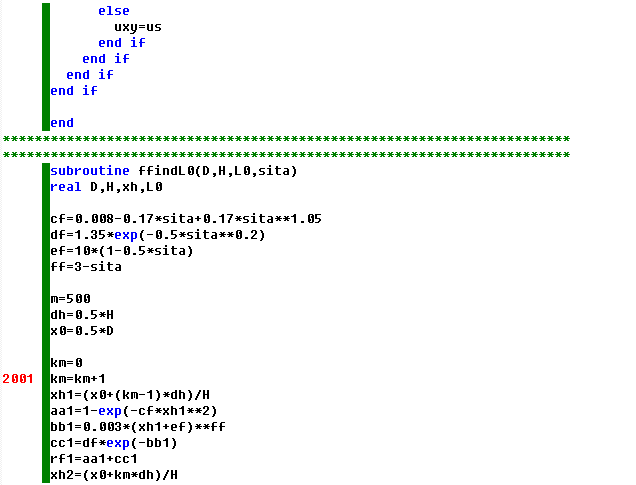


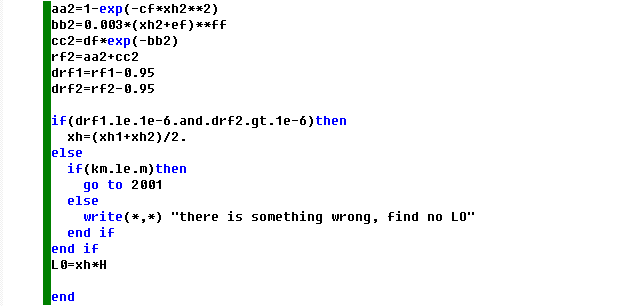


Figure S2 (End).
